# Vaccination and nutritional status of children in Karawari, East Sepik Province, Papua New Guinea

**DOI:** 10.1371/journal.pone.0187796

**Published:** 2017-11-09

**Authors:** Louis Samiak, Theophilus I. Emeto

**Affiliations:** 1 Department of Public Health, School of Medicine and Health Sciences, University of Papua New Guinea, Port Moresby, Papua New Guinea; 2 Public Health and Tropical Medicine, College of Public Health, Medical and Veterinary Sciences, James Cook University, Townsville, Australia; Public Health England, UNITED KINGDOM

## Abstract

Delivery of health care services to rural and remote populations in Papua New Guinea (PNG) is problematic. This is mainly due to difficulties with transportation and communication. Hence, the children in this region of PNG are likely to be at risk of malnutrition compounded by inadequate vaccination that may predispose them to preventable diseases. This study was conducted to determine the vaccination and nutritional status of children less than 5 years old in the remote and rural Karawari area of PNG. 105 children were included in the study, of whom 55% were male and 45% female. The mean age of children included in the study was 32.6 months. Their age, height, and weight by gender was not significantly different. Overall, 85% of children had incomplete vaccination. However, children above the median age of 32 months (34%) were more likely to be fully vaccinated for their age, χ2 (1) = 23.294, p < 0.005. In addition, 25% of children were below the -1 SD (Z-scores) for weight-for—height, 33% below the -1 SD for weight-for-age, and 25.5% below the -1 SD for height-for-age compared to WHO standards.

A large proportion of children had poor nutrition status and lack protection from vaccine preventable diseases. This study recommends that the government should introduce a surveillance system for detecting issues of importance to the rural majority. We also recommend that the PNG government reopen the nearby health centre, and/ or establish new facilities within the region, with adequately trained and compensated staff.

## Introduction

Delivery of health care services to rural and remote population are hampered by access due to inadequate transportation and communication [1, 2]. Hence services are concentrated in easily accessible urban areas, such as capital cities and towns [1]. Additionally, there are not enough health professionals to provide these services [1, 3]. Many rural communities are caught in a ‘*poverty-ill health-low productivity downward spiral’* [1].

Children in rural and remote areas within developed and developing countries have poor vaccination and nutritional status [4, 5]. Thus, they suffer from growth and developmental issues and are more at risk of dying in the first five years of life [5–8]. Vaccination is the most effective public health intervention against vaccine preventable disease and has saved millions of children’s lives [9]. Many parents however, have failed to realise the advantages of vaccination, and thus have not benefitted fully from health care services [6]. In Papua New Guinea (PNG), the majority of people live in rural areas [3, 10–12]. Health facilities are located in difficult to reach areas, with little support from government entities [13]. Wide disparities and regional differences are common, and most of the resources are concentrated in urban areas [12, 13]. The poor socioeconomic conditions and geographical terrain linked to health facilities are some contributing factors to low vaccination coverage [6]. Partially vaccinated children are at risk of resurgence of epidemics of vaccine preventable diseases from communities [6]. It is also common to have low vaccination coverage in areas where outreach patrols are either non-existent or inconsistent [14]. Consequently, children miss essential vaccinations that provide protection.

The nutritional status of children is a major predictor of child survival [15, 16]. Children require adequate food to grow and develop appropriately. At least three or four meals a day is recommended for growing children. Regrettably, malnutrition is widespread and common in rural communities particularly in children under five years of age [6, 17]. Food scarcity is common in the rural areas of PNG, which leads to under nutrition [11, 14]. Parents also misunderstand the rationale for having a balanced diet, which exacerbates the problem [11, 18]. In addition, the male child is often involved in activities outside the home, having had little food while the female child is engaged in household chores and mind younger siblings [14, 19], a notion that is culturally acceptable. Due to the regional and seasonal variation in outbreaks of malnutrition [17], there is the need for a surveillance system to monitor the degree of under nutrition [20]. Currently, there is none available within these regions of PNG.

Malnutrition remains a key global health issue within PNG. Anthropometric measures (commonly height and weight) are often used to assess nutritional status in children [21]. This is mainly due to the relative ease of obtaining these measurements. This study was conducted to determine the vaccination and nutritional status of children less than five years in the remote and rural Karawari area of PNG.

## Methods

This was a cross-sectional study carried out in April 2012. Protocol used for obtaining data was an adaptation of that published by Lapham et al. [22] and Meia et al. [23].

### Study setting

**Fig 1:**
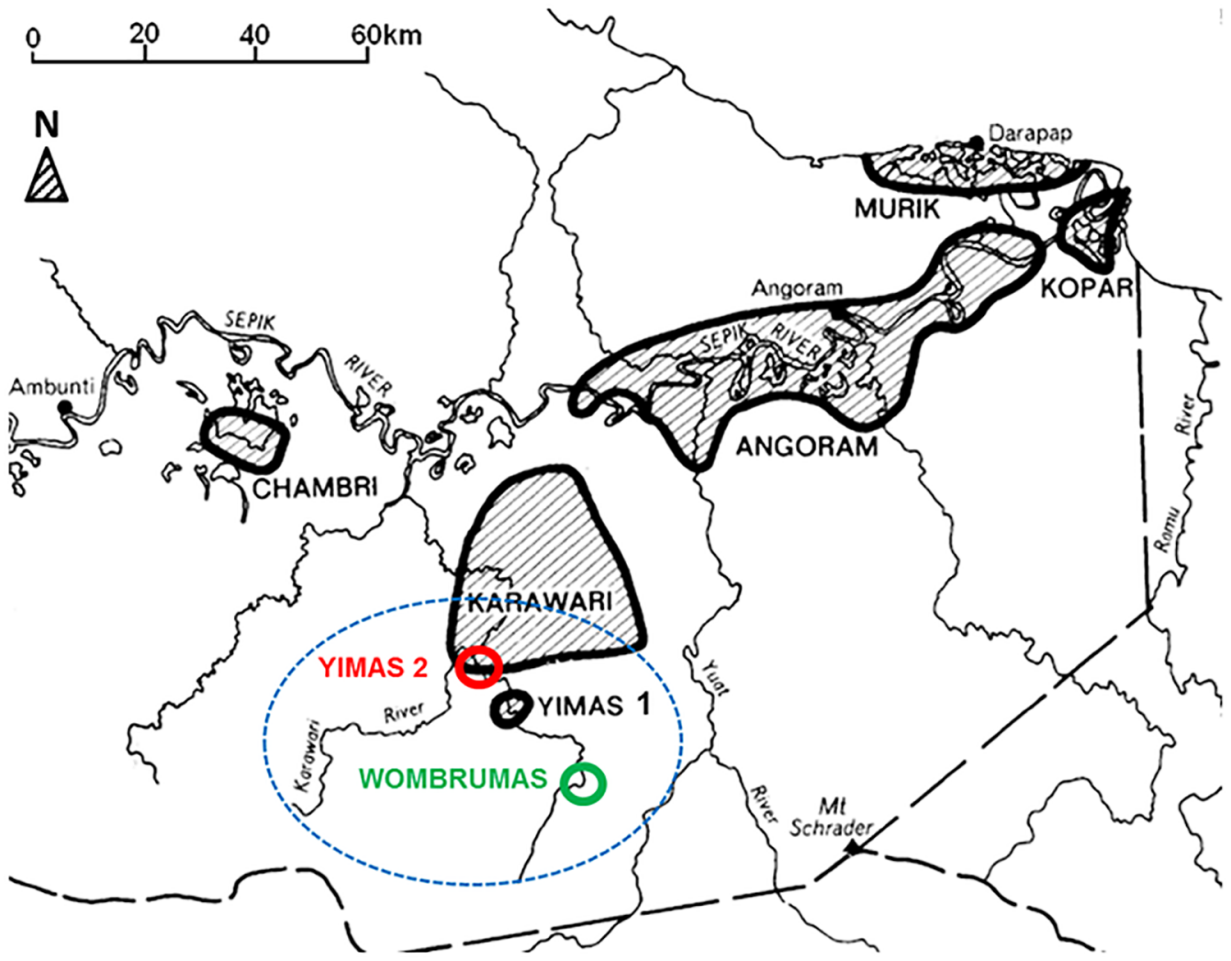
Map of Arafundi River and the study location, within Karawari local level government, East Sepik, PNG. Figures shows the study location within East Sepik (blue circle), modified from Wikimedia Commons, NorPondo [40].

Papua New Guinea lies north of Australia, with a population of about 7.2 million people [18]. Approximately 87% of this population are based in rural or remote areas [10]. East Sepik Province in PNG, with over 450,530 people [18], has six districts; Angoram which is one of them has six local level governments, including Karawari. The Arafundi River area is part of the Karawari local level government, which borders Enga and Madang Provinces (Fig 1). The nearest health centre to the study setting is almost six hours boat ride downstream. Each village has Village Health Volunteers (VHV), who had been trained several years ago, but are unable to provide adequate health care as they lack medical supplies. The government acknowledges, but does not recognize the VHV, and do not provide ongoing training support [10].

### Study population

The participants are children of third generation semi-nomadic tribes who settled in these locations over 100 years ago. The vaccination status and the anthropometric parameters of 105 children within three villages along the Arafundi River in the Karawari region were collected. Verbal informed consent was obtained prior to data collection from parents or caregivers, after the importance of the study was explained, as it was the most convenient cultural approach for participants. The University of Papua New Guinea human ethics committee provided approval for this study including the verbal informed consent from participants.

### Inclusion and exclusion criteria

All children aged not more than 60 months with available health record books were included. Children born before February 2007 and after February 2012 and those without health record books were excluded from the study.

### Measurements

Data from all accessible and available children who met the study inclusion criteria in the villages were included (105 out of 125 children present in the villages). Twenty (16%) of the available 125 children were excluded as they had no health record book. The following information Sex, date of birth, age, vaccination status, and last date of vaccination were obtained from child health record books. In PNG, children are expected to be fully immunised at 12 months of age [11]. The normal vaccination program schedule is: BCG and Hep B given at birth, DTP, Polio, HiB, Hep B given at 1, 2, 3 months and multiple doses of Measles vaccine given from 6 months. Full vaccination was defined as having received all recommended vaccines for that age. Incomplete vaccination was defined as any child that had missed at least two recommended vaccines for that age as per the schedule. The height or length data from children below the age of 60 months of age was taken to the nearest 0.1 cm. The height was measured with a tape measure mounted on a hard surface, on bare foot, for those over 12 months. The length of children less than 24 months or not walking was taken in place of the height using a designated wooden infantometer in a recumbent position. Weight was recorded using an ordinary foot scale, which was zeroed after every fifth participant, and was recorded to the nearest 0.1 kg. All measurements were taken in duplicates and the mean presented.

Based on standardised age-and gender- specific growth references [21, 24], we computed the growth and general nutritional status of children. We employed the weight-for-age Z-scores (WAZ), weight-for-height Z-scores (WHZ), and height-for-age Z-scores (HAZ based on the World Health Organisation (WHO) charts(24). Based on WHZ, ‘acute malnutrition’ was defined as global malnutrition (< -2 Z-score), moderate malnutrition (< -2 Z-score and >= -3 Z-score), and severe malnutrition (< -3 Z-scores). Based on WAZ, ‘underweight’ was defined as underweight (< -2 Z-score), moderate underweight (< -2 Z-score and >= -3 Z-score), and severe underweight (< -3 Z-scores). Based on HAZ, ‘stunting’ was defined as stunting (< -2 Z-score), moderate stunting (< -2 Z-score and >= -3 Z-score), and severe stunting (< -3 Z-scores)(24). Nutritional status was further classified as “normal”, Z-scores > -2 Z-scores for WAZ, WHZ, and HAZ, and < -2 Z-scores for WAZ (underweight), HAZ (stunted), and WHZ (malnourished).

### Data analysis

De-identified data was analysed using SPSS version 23.0 (IBM, Chicago, IL, USA) and ENA Software Version 2011 for SMART. Numerical data were assessed for normality and the appropriate descriptive statistics reported. Categorical data are presented as numbers and percentages. Associations between vaccination status and gender and between vaccination status and age groups were tested using the Chi-squared test of independence (or Fisher’s exact test). Differences between age, height and weight based on vaccination status were assessed employing the Independent-samples t-test. The Pearson’s correlation coefficient was use to assess the strength of association between age, height and weight. Data are presented as mean ± standard deviation unless otherwise stated.

ENA software was used for the nutritional status analysis. This software applies the Box-Cox Power Exponential (BCPE) methods to obtain smoothened centile curves by cubic splines [25]. Conventional outliers with a ± 3 standard deviation (SD) of WHZ from the observed WHZ mean were not excluded from the analysis. This is typical of data obtained in similar resource limited areas [26]. One child had missing data on height and was excluded from nutritional status analysis. WHO recommended cut-offs of HAZ, WAZ of <-6 to 5 were applied, since these extreme values were likely due to data entry errors [24]. Odds ratio (OR) and 95% confidence interval (CI) were derived for the relationship between WHZ, WAZ, and HAZ with vaccination status. All statistical tests were performed using two-tailed comparisons with 95% level of confidence.

## Results

A total of 105 children were included in the study, 58 females (55%) and 47 males (45%). One child had no data for height, and was excluded from the WHZ and HAZ analysis. The distribution of age and Sex of children included in the study are summarised in Table 1. Overall, age ranged from 3 months to 59 months, with a mean of 31.6 ± 15.7. Weight ranged from 5.2 to 17.7 kg, with a mean of 11.2 ± 3.0. Height ranged from 40 to 106 cm with a mean of 84.7 ± 12.0.

**Table 1:** Distribution of age and sex of children in the study.

Age (months)*	Girls	Boys	Total	Ratio
	n (%)	n (%)	n (%)	Girl:Boy
**0–10**	3 (27.3)	8 (72.7)	11(10.7)	0.4
**11–20**	9 (50.0)	9 (50.0)	18 (17.5)	1.0
**21–30**	10 (50.0)	10 (50.0)	20 (19.4)	1.0
**31–40**	16 (76.2)	5 (23.8)	21 (20.4)	3.2
**41–50**	11 (52.4)	10 (47.6)	21 (20.4)	1.1
**51–59**	7 (58.3)	5 (41.7)	12 (11.7)	1.4
**Total**	56 (54.4)	47 (45.6)	103 (100)	1.2

*Note children aged greater than 59 months were excluded.

A comparison of the age, height and weight for children by gender was determined by the independent samples t-test. There was homogeneity of variances, as assessed by Levene's test for equality of variances for height and age (*p* > 0.05) but the assumption was violated for weight (*p* < 0.05). Girls height in cm (85.9 ± 10.6), age in months (34.00 ± 14.42), and weight in kg (11.3 ± 2.7) were not statistically different to boys height in cm (83.3 ± 13.3), age in months (28.6 ± 16.9), and weight in kg (11.0 ± 3.4), *p* > 0.05 respectively.

### Relationship between vaccination status and anthropometric parameters

#### Vaccination status

The vaccination status of children in this study are summarised below. Sixteen children (15%) were fully vaccinated and 89 children (85%) had incomplete vaccination. When stratified by gender, 8 girls (14%) out of 58, and 8 boys (17%) out of 47 were fully vaccinated. There was no statistically significant association between gender and vaccination status, χ^2^ (1) = 0.209, *p > 0*.*05*. When age was categorised based on the median age below 32 months and from 32 months and above. Age group was significantly associated with vaccination status, χ^2^ (1) = 23.294, *p < 0*.*005*. This association was moderately strong, φ = -0.471, *p* < 0.005. Children above the median age of 32 months (34%) were more likely to be fully vaccinated. No child (0%) below the median age was fully vaccinated.

#### Nutritional status

**Fig 2:**
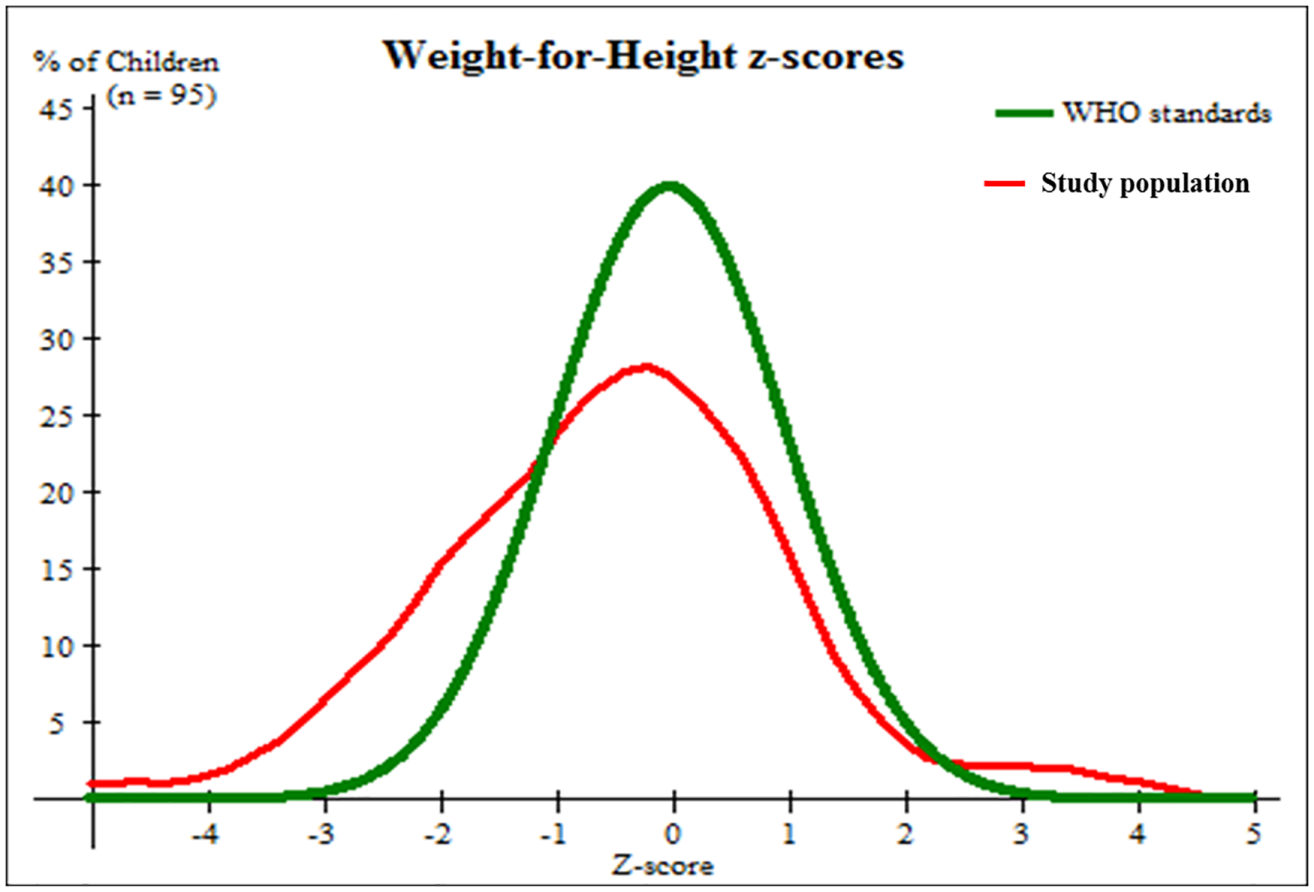
Weight-for-height chart of children aged five or less within the Karawari region compared to WHO standards. Figures shows that 25% children were below -1 SD (Z- scores), 17% children were below -2 SD and 7% children were below -3 SD. The relation between weight and height of the children was statistically significant(r = 0.864 and p<0.001).

We found that the prevalence of wasting (WHZ less than 2SD below the mean) was higher than the WHO standards (Fig 2). Our data shows that 25% children were below the -1 SD (Z- scores), 17% were below the -2 SD and 7% below the -3 SD. The weight and height of the children in the study were statistically significantly correlated, r = 0.864, and p<0.001. The overall prevalence of acute malnutrition based on WHZ was 15.8%. Global malnutrition in boys (16.7%) was higher than for girls (15.1%). Boys were also more likely to have severe malnutrition (9.5%) than girls (1.9%). Girls were more likely to have moderate malnutrition (13.2%) than boys (7.1%). However, these nutritional were not statistically significantly different between boys and girls (Table 2).

**Table 2:** Prevalence of acute malnutrition based on weight-for-height z-scores and by sex.

Prevalence	All (n = 95)	Girls (n = 53)	Boys (n = 42)
	n (%, 95% C.I)	n (%, 95% C.I)	n (%, 95% C.I)
**Global malnutrition (<-2 z-score)**	15 (15.8%, 9.8–24.4)	8 (15.1%, 7.9–27.1)	7 (16.7%, 8.3–30.6)
**Moderate malnutrition (<-2 z-score and >= -3 z-score)**	10 (10.5%, 5.8–18.3)	7 (13.2%, 6.5–24.8)	3 (7.1%, 2.5–19.0)
**Severe malnutrition (<-3 z-score)**	5 (5.3%, 2.3–11.7)	1 (1.9%, 0.3–9.9)	4 (9.5%, 3.8–22.1)

Abbreviations: % = percentages, C.I = confidence interval, n = sample size

**Fig 3:**
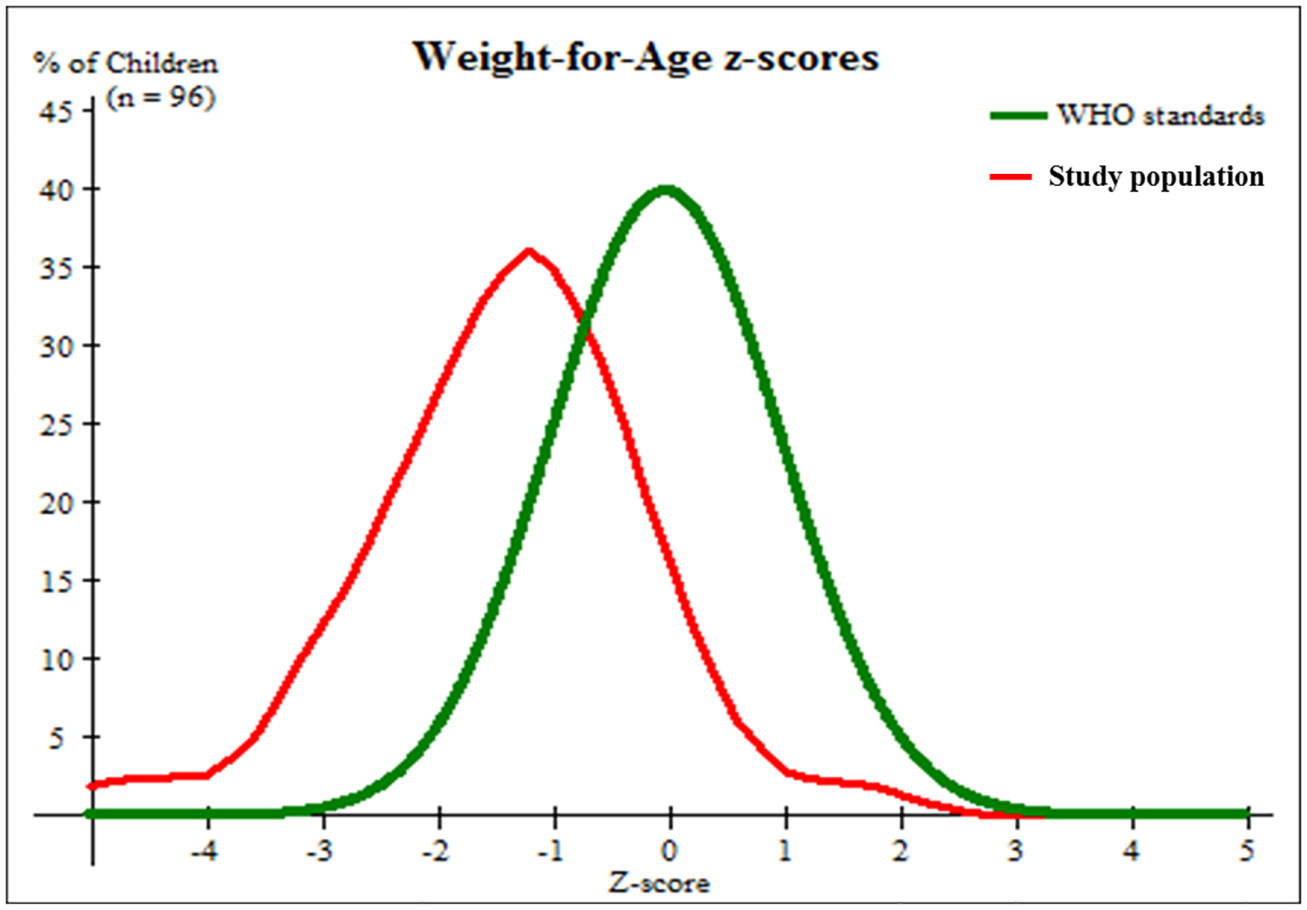
Weight-for-age chart of children aged five or less within the Karawari region compared to WHO standards. Figures shows the nutritional status of children on the basis of weight-for-age; 33% of the children were below -1 SD, 33.5% were below -2 SD, and 11.5% below -3 SD. Correlation between weight and age is statistically significant (r = 0.861 and p<0.001).

In addition, an assessment of the nutritional status of children on the basis of WAZ reveals that 33% of children were below -1 SD, 33.5% below -2 SD, and 11.5% below -3 SD (Fig 3). Weight and age of the children were significantly correlated, r = 0.861, p<0.001. Overall prevalence of underweight based on weight-for-age was 26%. Boys were more likely than girls to be underweight (33.3% vs20.4%), moderately underweight (21.4% vs 14.8%), and severely underweight (11.9% vs 5.6%), although these differences were not statistically significant (Table 3).

**Table 3:** Prevalence of underweight based on weight-for-age z-scores by sex.

Prevalence	All (n = 96)	Girls (n = 54)	Boys (n = 42)
	n (%, 95% C.I)	n (%, 95% C.I)	n (%, 95% C.I)
**Underweight (<-2 z-score)**	25 (26.0%, 18.3–35.6)	11 (20.4%, 11.8–32.9)	14 (33.3%, 21.0–48.4)
**Moderate underweight (<-2 z-score and >= -3 z-score)**	17 (17.7%, 11.4–26.5)	8 (14.8%, 7.7–26.6)	9 (21.4%, 11.7–35.9)
**Severe underweight (<-3 z-score)**	8 (8.3%, 4.3–15.6)	3 (5.6%, 1.9–15.1)	5 (11.9%, 5.2–25.0)

Abbreviations: % = percentages, C.I = confidence interval, n = sample size

**Fig 4:**
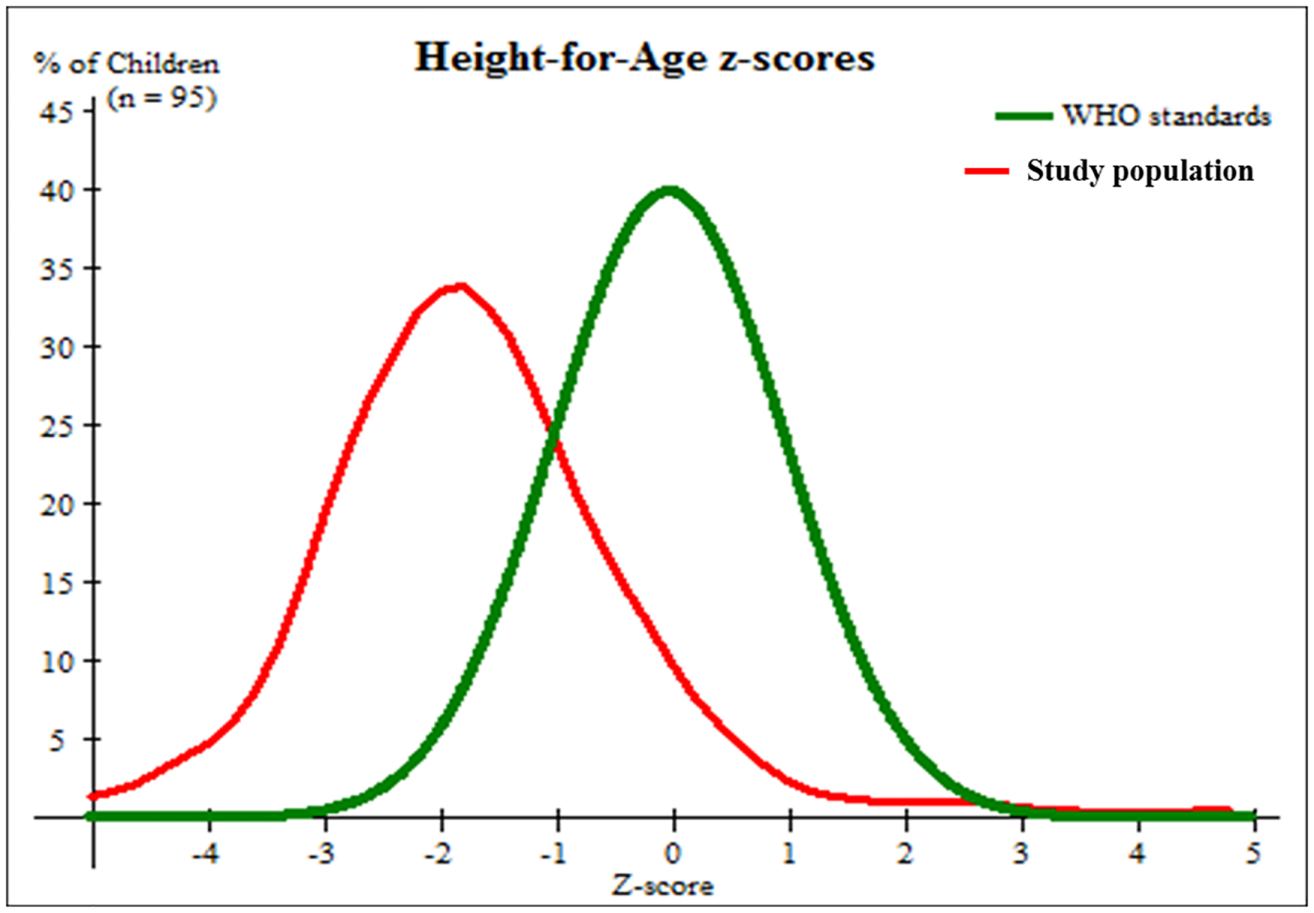
Height-for-age chart of children aged five or less within the Karawari region compared to WHO standards. Figures shows the nutritional status of children according to the height-for-age, 25.5% were below -1 SD, 34% were below -2 SD, and 20% were below -3 SD. The height and age of the children had statistically significant relation (r = 0.882 and p<0.001).

We assessed the nutritional status of the children based on the HAZ (Fig 4). Data shows that 25.5% of children were below the -1 SD, 34% were below -2 SD, and 20% were below the -3 SD. The height and age of the children was statistically significant associated, r = 0.882, p<0.001. Overall prevalence of stunting based on HAZ was 45.3% (Table 4). There was no statistical difference in the proportion of stunting in girls (49.1%) compared to boys (40.5%).

**Table 4:** Prevalence of stunting based on height-for-age z-scores and by sex.

Prevalence	All (n = 96)	Girls (n = 54)	Boys (n = 42)
	n (%, 95% C.I)	n (%, 95% C.I)	n (%, 95% C.I)
**Stunting (<-2 z-score)**	43 (45.3%, 35.6–55.3)	26 (49.1%, 36.1–62.1)	17 (40.5%, 27.0–55.5)
**Moderate stunting (<-2 z-score and >= -3 z-score)**	32 (33.7%, 25.0–43.7)	20 (37.7%, 25.9–51.2)	12 (28.6%, 17.2–43.6)
**Severe stunting (<-3 z-score)**	11 (11.6%, 6.6–19.6)	6 (11.3%, 5.3–22.6)	5 (11.9%, 5.2–25.0)

Abbreviations: % = percentages, C.I = confidence interval, n = sample size

Finally, to adjust for the potential confounding effect of age, we calculated the odds ratio (OR) of a child having full vaccination based on whether a child was underweight, stunted or malnourished. Underweight children (3, 11.5%) are less likely to be fully vaccinated compared to children within normal weight ranges (13, 16.5%), OR = 0.662 (95% CI, 0.173 to 2.534). Children with stunting (6, 13.6%) are less likely to be fully vaccinated compared to children without stunting (10, 16.4%), OR = 0.805 (95% CI, 0.269 to 2.409). Malnourished children (1, 6.3%) are less likely to be fully vaccinated compared to children with normal nutrition (15, 16.9%), OR = 0.329 (95% CI, 0.040 to 2.683). However, none of trends were statistically significant.

## Discussion

This study assessed the nutritional and vaccination status of children within three villages in the Karawari area of PNG. The area is plagued by accessibility issues and poverty, and suffers from limited health care services. Residents in the region have had inadequate health care service and irregular maternal-child health provision since the only health facility closed twenty years ago. Since the introduction of the Expanded Programme on Immunisation (EPI) in 1981, the health department in PNG have introduced the slogan “all children under one year must be immunised” [11]. However, the reality is that many health workers are unable to implement this mandate, mainly because of the difficulties associated with assessing and providing health care in the various remote communities. Hence, about 30% of health facilities within PNG are non-functional. The results of this study demonstrate that many children are not fully immunised. As protection from diseases is low, there is a very high risk of an outbreak of vaccine-preventable disease in the community [6, 27]. Vaccination is advocated as an effective public health measure [28], but has not been administered as recommended to the children in this area. Most children have incomplete vaccination status with long gaps between scheduled vaccinations, primarily due to a lack of vaccines availability [29] as a consequence of limited health care services. The incomplete vaccination statuses of the children denies them of an essential public health measure. Unfortunately, we were unable to stratify incomplete vaccination, by the vaccination type, which is one of the limitations of our study.

Nutritional status of children is a strong determinant of their future wellbeing and survival [16, 30–32]. Deficiencies occur most commonly in children less than 5 years old, and may cause serious diseases and even death [5, 33]. Malnutrition is at the core of conditions that contribute to diseases [33]. This study found that the percentage of under-nutrition was 26%, stunting was 45.3%, and wasting was 15.8% which is slightly higher than the national average but similar to other rural and remote areas in PNG such as the Momase region [11]. In addition, this finding is below the WHO standards but consistent with findings in similar resource limited settings [34–36]. Although seasonal variation in the availability of the staple food (sago and fish) [37], may have impacted on the nutritional statuses reported in this study.

Malnutrition is an indication of food insecurity in a community [26], and is influenced by the degree of development in the area [32]. As development progresses, economic activities increase, and the general standard of living improves in turn.

Inadequate and inappropriate food consumption has been reported to contribute to stunting and poor linear growth [38]. Not surprisingly, a significant number of children in this study exhibit stunted growth as per WHO guidelines, which may be an indication of an inadequacy in their diet, although we did not examine feeding patterns. Stunting begins in the first 1000 days [39], and causes serious life-long cognitive and developmental issues [17, 18]. Therefore, further studies are recommended to ascertain whether inadequate food intake is linked to the poor nutritional status of children in remote and rural PNG. Finally, data from this study shows that children that with a compromised nutritional status are less likely to be fully vaccinated. Although not a significant finding, it is nevertheless a concerning trend, as this could have lasting health and wellbeing implication.

### Conclusion

There is large number of children within our study region with incomplete vaccination and a significant proportion are malnourished. Consequently, there is low protection from vaccine-preventable diseases, posing a risk of an outbreak of disease in the community.

### Recommendations

This study recommends that the responsible PNG stakeholders should ensure children are fully vaccinated and thus protected from vaccine preventable diseases. We also recommend the establishment of a surveillance system to monitor nutritional status of vulnerable children. In the long term, the PNG government should reopen the nearby health centre and/ or establish new facilities within the region, with adequately trained and compensated staff. Other alternatives to consider are the use of Provincial authorities and private-public partnership arrangement, and intervention programs targeted at women and children. These strategies will facilitate the eradication of malnutrition and vaccine-preventable disease outbreak, which are a serious problem in PNG.

## Supporting information

S1 Data Raw data (Samiak and Emeto 2017_Needs data.xlsx) underlying the findings described in the manuscript available online. (XLSX)
