# Correction: Vaccination and nutritional status of children in Karawari, East Sepik Province, Papua New Guinea

**DOI:** 10.1371/journal.pone.0193631

**Published:** 2018-02-23

**Authors:** The PLOS ONE Staff

## Notice of Republication

This article was republished on November 20, 2017 to correct errors in figures that were introduced during the typesetting process. The publisher apologizes for the errors. Please download this article again to view the correct figures. The originally published, uncorrected article and the republished article with corrected figures are also provided here for reference.

## Supporting information

S1 File Originally published, uncorrected article. (PDF)

S2 File Republished, corrected article. (PDF)
